# Advanced Oxidation Protein Products Contribute to Chronic-Kidney-Disease-Induced Adipose Inflammation through Macrophage Activation

**DOI:** 10.3390/toxins15030179

**Published:** 2023-02-26

**Authors:** Nanaka Arimura, Hiroshi Watanabe, Hiromasa Kato, Tadashi Imafuku, Takehiro Nakano, Miyu Sueyoshi, Mayuko Chikamatsu, Kai Tokumaru, Taisei Nagasaki, Hitoshi Maeda, Motoko Tanaka, Kazutaka Matsushita, Toru Maruyama

**Affiliations:** 1 Department of Biopharmaceutics, Graduate School of Pharmaceutical Sciences, Kumamoto University, 5-1 Oe-honmachi, Chuo-ku, Kumamoto 862-0973, Japan; 2 Department of Nephrology, Akebono Clinic, Kumamoto 862-0973, Japan

**Keywords:** advanced oxidation protein products, albumin, chronic kidney disease, adipose inflammation, macrophage migration, macrophage polarization

## Abstract

Fat atrophy and adipose tissue inflammation can cause the pathogenesis of metabolic symptoms in chronic kidney disease (CKD). During CKD, the serum levels of advanced oxidation protein products (AOPPs) are elevated. However, the relationship between fat atrophy/adipose tissue inflammation and AOPPs has remained unknown. The purpose of this study was to investigate the involvement of AOPPs, which are known as uremic toxins, in adipose tissue inflammation and to establish the underlying molecular mechanism. In vitro studies involved co-culturing mouse-derived adipocytes (differentiated 3T3-L1) and macrophages (RAW264.7). In vivo studies were performed using adenine-induced CKD mice and AOPP-overloaded mice. Fat atrophy, macrophage infiltration and increased AOPP activity in adipose tissue were identified in adenine-induced CKD mice. AOPPs induced MCP-1 expression in differentiated 3T3-L1 adipocytes via ROS production. However, AOPP-induced ROS production was suppressed by the presence of NADPH oxidase inhibitors and the scavengers of mitochondria-derived ROS. A co-culturing system showed AOPPs induced macrophage migration to adipocytes. AOPPs also up-regulated TNF-α expression by polarizing macrophages to an M1-type polarity, and then induced macrophage-mediated adipose inflammation. In vitro data was supported by experiments using AOPP-overloaded mice. AOPPs contribute to macrophage-mediated adipose inflammation and constitute a potential new therapeutic target for adipose inflammation associated with CKD.

## 1. Introduction

Chronic kidney disease (CKD) patients often experience metabolic diseases such as insulin resistance and dyslipidemia, which are a risk factor for cardiovascular disease and can shorten life expectancy [1,2]. Recently, fat atrophy, which is a decrease in whole-body adipose tissue, has been found to be associated with the pathogenesis of metabolic symptoms in CKD [3,4].

Adipose tissue plays an important role in maintaining the homeostasis of systemic energy metabolism by storing excess energy and generating free fatty acids through lipolysis as required. Therefore, adipose tissue dysfunction results in a reduced fat storage capacity and ectopic lipid deposition in tissues such as the liver and skeletal muscle. This ectopic lipid deposition contributes to the development of metabolic diseases [5,6]. Although decreased fat mass is associated with increased mortality in hemodialysis patients [7], the underlying mechanism through which fat atrophy occurs during CKD has not been determined.

Fat atrophy occurs via increased lipolysis, which is related to dysfunctional adipose tissue. Inflammatory cytokines (TNF-α and IL-6) released from adipocytes are thought to be implicated in this lipolysis [8,9,10]. In general, macrophage infiltration is involved in the chronic inflammation of adipose tissue, and macrophage infiltration occurs with the increased expression of the monocyte chemotactic factors (MCP-1, etc.) associated with increased oxidative stress in adipose tissue [11,12]. The TNF-α released from infiltrating macrophages induce inflammatory cytokine production in adipocytes, causing chronic inflammation throughout the adipose tissue [13]. Macrophage accumulation in adipose tissue, adipose inflammation and ectopic lipid deposition in the liver and skeletal muscle have been reported in both CKD patients and CKD animal models [14,15]. However, the molecular mechanisms underlying the induction of adipose inflammation in CKD remain unclear.

The level of serum advanced oxidation protein products (AOPPs), which are uremic toxins, increases during CKD pathology resulting in oxidative stress [16]. AOPPs are oxidatively modified proteins that are generated via a reaction with chlorinated oxidants such as the hypochlorous acid (HOCl) produced via myeloperoxidase in neutrophils. The HOCl oxidatively modifies serum proteins (mostly albumin) via carbonylation and the formation of dityrosine [17]. Previously, AOPPs have been reported to increase oxidative stress, which contributes to the pathogenesis of renal tubular disorders, osteoporosis, and Crohn’s disease [18,19,20]. Recently, we also reported that AOPPs are involved in the pathogenesis of CKD-induced sarcopenia through the enhanced production of reactive oxygen species (ROS) via the CD36/NADPH oxidase pathway in muscle cells [21].

However, the relationship between fat atrophy/adipose tissue inflammation and AOPPs has remained unknown. The purpose of this study was to determine the involvement of AOPPs in adipose tissue inflammation and to establish its molecular mechanism. First, using adenine-induced CKD mice, we evaluated fat atrophy, macrophage infiltration and the activity of AOPPs in adipose tissue. Next, using mouse-derived 3T3-L1 adipocytes and a mouse macrophage-like cell line (RAW264.7), we investigated the molecular mechanisms of adipose inflammation induced by AOPPs. Finally, we examined AOPP-induced adipose tissue inflammation using AOPP-overloaded mice.

## 2. Results

### 2.1. Evaluation of Adipose Tissue in Adenine-Induced CKD Mice

**Figure 1:**
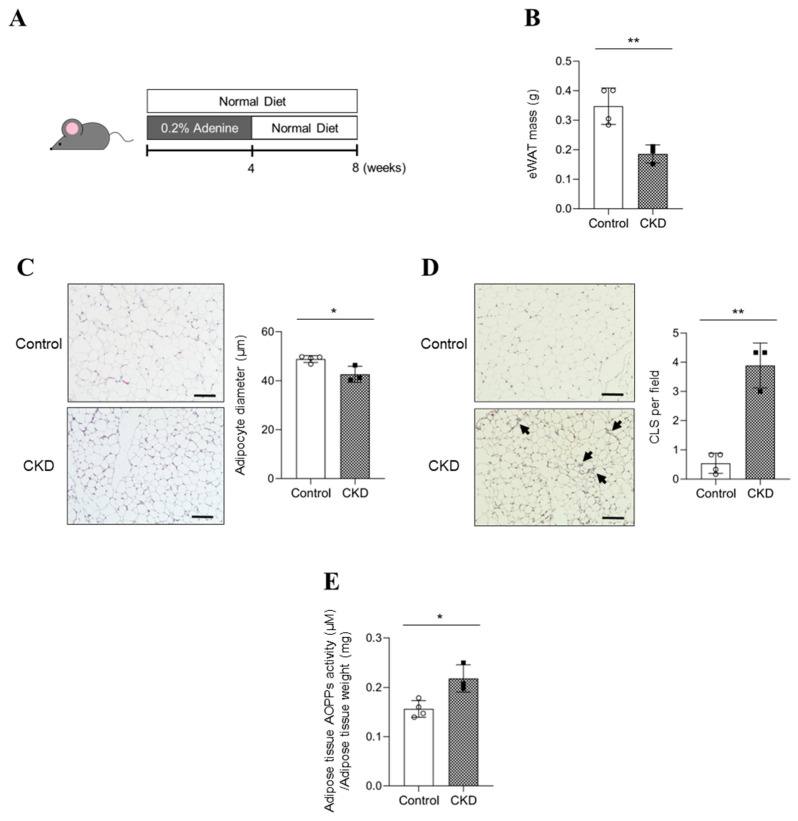
Evaluation of adipose tissue in adenine-induced CKD mice. (**A**) Experimental protocol for the evaluation of adipose tissue in adenine-induced CKD mice. (**B**) The weight of epididymal white adipose tissue (eWAT) in control and CKD mice. (**C**) Paraffin sections of eWAT were stained with hematoxylin and eosin to determine the diameters of adipocytes. Original magnifications: ×200. Scale bars represent 100 μm. (**D**) Representative images of immunohistological staining of F4/80 and quantification of crown-like structures (CLSs) in eWAT from control and CKD mice. The black arrow indicates CLSs. Original magnifications: ×200. Scale bars represent 100 μm. (**E**) AOPP levels were measured in eWAT of control and CKD mice. Data are expressed as the mean ± SEM (n = 3–4). * *p* < 0.05, ** *p* < 0.01 compared with control.

Figure 1A showed the experimental protocol for the evaluation of adipose tissue in adenine-induced CKD mice. Regarding the validity of CKD model mice, the renal function (blood urea nitrogen: BUN, serum creatinine: SCr) and body weight are shown in Table S2. As the representative fat tissue, epididymal white adipose tissue (eWAT) was evaluated. The weight of eWAT was significantly decreased in CKD mice by comparison with the control mice (Figure 1B). H&E staining revealed that the adipocyte diameter was significantly reduced in the CKD mice (Figure 1C). F4/80 immunostaining showed macrophage infiltration in the adipose tissue of CKD mice (Figure 1D). Next, we investigated whether AOPPs were involved in the observed adipose tissue loss and macrophage infiltration by evaluating AOPP activity in eWAT. A significant increase in AOPP activity was observed in the eWAT of CKD mice compared to the control mice (Figure 1E). These data suggested that AOPPs may be involved in the observed fat atrophy and macrophage infiltration in CKD.

### 2.2. Molecular Mechanisms of ROS Production and Macrophage Infiltration by AOPPs

**Figure 2:**
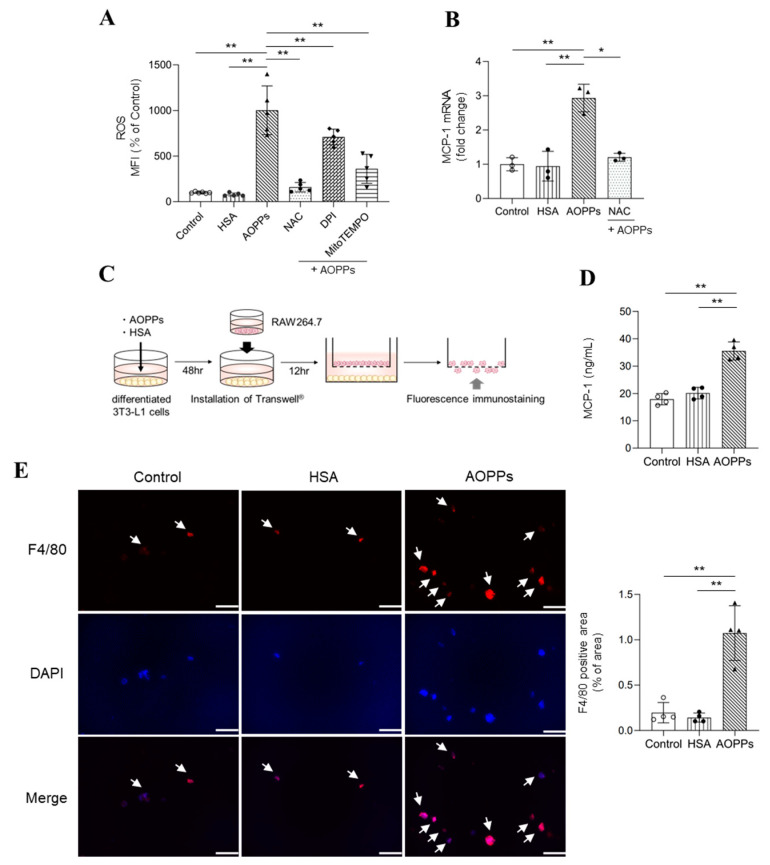
The mechanism for AOPP-induced ROS production and macrophage infiltration. (**A**) Effect of AOPPs (100 μM) on ROS production in differentiated 3T3-L1 cells. Differentiated 3T3-L1 cells were treated with CM-H2DCFDA in D-PBS for 30 min. After the removal of the D-PBS, cells were treated with AOPPs and incubated for a further 60 min. Fluorescence intensity was measured at an excitation wavelength of 485 nm and at an emission wavelength of 535 nm. In the inhibitor experiments, CM-H2DCFDA in D-PBS was removed and each inhibitor was then added. After incubating for 30 min, the cells were treated with AOPPs and incubated for 60 min in the presence of each inhibitor. Antioxidant (N-acetyl-L-cysteine: NAC (2 mM)), NADPH oxidase inhibitor (diphenylene iodonium: DPI (50 μM)) and mitochondrial ROS scavenger (MitoTEMPO (25 μM)) were used. (**B**) Effect of AOPPs on mRNA expression of MCP-1 in differentiated 3T3-L1 cells 12 h after treatment with 100 μM AOPPs. NAC (2 mM) was added 1 h before AOPP treatment. (**C**) Experimental protocol for the evaluation of macrophage migration to adipocytes in the presence of AOPPs using a Transwell^®^ system. Differentiated 3T3-L1 cells were treated with 100 μM AOPPs. At 48 h after AOPP treatment, Transwell^®^ inserts seeded with RAW264.7 cells were introduced and co-cultured for 12 h. (**D**) The level of MCP-1 protein in the medium of the lower chamber was measured using ELISA at 60 h after treatment with 100 μM AOPPs. (**E**) After co-culture for 12 h, macrophages that migrated to the lower layer of the membrane were fluorescently immunostained using anti-F4/80 antibody. The white arrow indicates F4/80 positive macrophage. Original magnifications: ×200. Scale bars represent 100 μm. Data are expressed as the mean ± SEM (n = 3–6). * *p* < 0.05, ** *p* < 0.01.

To determine the relationship between AOPPs and ROS production or macrophage infiltration, differentiated 3T3-L1 adipocytes were used in the study. Firstly, we evaluated the effect of AOPPs on ROS production. AOPPs significantly increased ROS production in adipocytes at a concentration of 100 μM AOPPs as observed in the serum of CKD patients (Figure 2A). In contrast, no significant increase in ROS was observed after treatment with HSA at the same protein level as that of AOPPs. Next, the mechanism of AOPP-induced ROS production was examined. NADPH oxidase and mitochondria are known to be the major sources of ROS production in adipocytes [22]. Therefore, we focused on NADPH oxidase and mitochondria using pharmacological inhibitors. AOPP-induced intracellular ROS was significantly suppressed by co-treatment with N-acetylcysteine (NAC), an antioxidant, diphenyleneiodonium chloride (DPI), an inhibitor of NADPH oxidase, and MitoTEMPO, a scavenger of mitochondria-derived ROS (Figure 2A). This observation suggests that AOPPs increase the level of ROS via NADPH oxidase and the mitochondria in adipocytes.

It has been reported that the increase in ROS and monocyte chemotaxis factor (MCP-1) expression is involved in adipocyte inflammation [23]. Therefore, we evaluated the effect of AOPPs on MCP-1 expression. Our results showed that MCP-1 expression was significantly increased after treatment with 100 μM AOPPs (Figure 2B). Moreover, increased MCP-1 expression was suppressed by co-treatment with NAC (Figure 2B). These data indicate that AOPPs induce MCP-1 expression through ROS production in adipocytes.

Next, we evaluated whether AOPPs promote the migration of macrophages into adipocytes using a co-culture system in a Transwell^®^. AOPPs were added to differentiated 3T3-L1 cells in the lower layer, and 48 h later, Transwell^®^ inserts seeded with a mouse macrophage cell line (RAW264.7 cells) were introduced and co-cultured for a further 12 h (Figure 2C). MCP-1 protein level in the lower culture medium was evaluated. The results showed that MCP-1 protein expression in the lower culture medium was significantly increased by treatment with AOPPs (Figure 2D). Macrophages migrating to the lower membrane were subsequently immunostained with anti-F4/80 antibody. A significant increase in the F4/80 fluorescence intensity was observed after incubation with AOPPs by comparison with the control culture or culture that underwent HSA treatment (Figure 2E). These results suggested that AOPPs enhance macrophage migration into adipose tissue by stimulating adipocytes to release MCP-1.

### 2.3. Molecular Mechanisms of AOPP-Induced Adipose Inflammation

**Figure 3:**
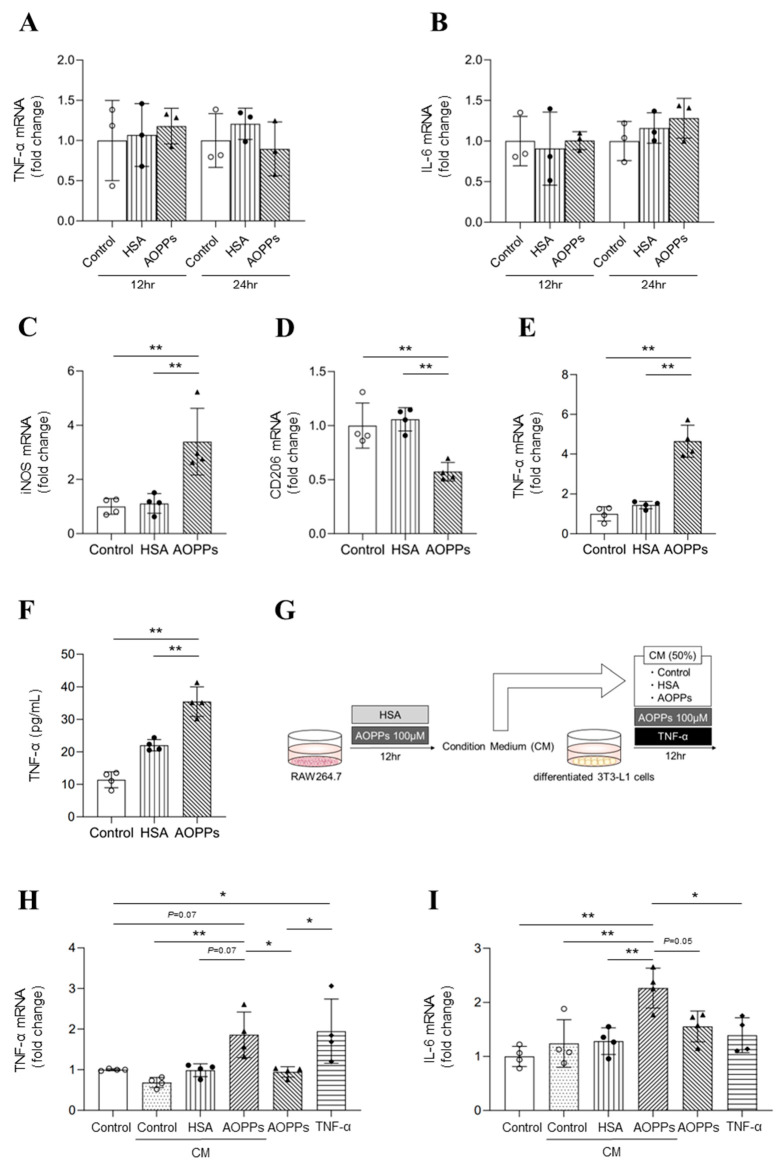
The mechanism for AOPP-induced adipocyte inflammation using differentiated 3T3-L1 cells and RAW264.7 cells. (**A**,**B**) The mRNA expression levels of (**A**) TNF-α and (**B**) IL-6 in differentiated 3T3-L1 cells at 12 or 24 h after treatment with 100 μM AOPPs were measured via real-time qPCR. (**C**,**D**) The mRNA expression levels of (**C**) iNOS and (**D**) CD206 in RAW264.7 cells at 12 h after treatment with 100 μM AOPPs were measured using real-time qPCR. (**E**,**F**) Effect of AOPPs on (**E**) TNF-α mRNA expression in RAW264.7 cells and (**F**) TNF-α protein level in the medium at 12 h after treatment with 100 μM AOPPs. (**G**) Experimental protocol to evaluate the involvement of AOPPs in macrophage-mediated adipose inflammation. RAW264.7 cells were treated with AOPPs, and the conditioned medium was collected 12 h later. The collected conditioned medium (50%) was used to treat differentiated 3T3-L1 cells for 12 h. (**H**,**I**) The mRNA expression of (**H**) TNF-α and (**I**) IL-6 in differentiated 3T3-L1 cells was evaluated 12 h after treatment with conditioned medium (50%). Differentiated 3T3-L1 cells were also incubated with 100 μM AOPPs or TNF-α (75 pg/mL) for 12 h. Data are expressed as the mean ± SEM (n = 3–4). * *p* < 0.05, ** *p* < 0.01.

To evaluate the adipose inflammatory state, the effects of AOPPs on inflammatory cytokine (TNF-α and IL-6) expression in differentiated 3T3-L1 cells were examined. No significant differences in TNF-α and IL-6 expression were observed at 12 and 24 h after HSA and AOPP treatment compared to the control (Figure 3A,B). Given that adipose tissue–macrophage interactions have been reported to contribute to adipose inflammation, we focused on the effect of AOPPs on adipose tissue–macrophage interactions. Firstly, we evaluated the effect of AOPPs on macrophage polarity. Specifically, AOPPs were added to RAW264.7, and then the expression of iNOS was evaluated using an M1 macrophage (pro-inflammatory) marker, with CD206 evaluated using an M2 macrophage (anti-inflammatory) marker. The results showed that iNOS mRNA expression was significantly increased after AOPP treatment compared to control cells or cells that underwent HSA-treatment (Figure 3C). By contrast, however, CD206 mRNA expression was significantly decreased in cells treated with AOPPs (Figure 3D). Treatment with AOPPs also significantly increased TNF-α mRNA expression in RAW264.7 cells and TNF-α protein expression in the culture medium (Figure 3E,F). These results indicate that AOPPs directly affect macrophages. Moreover, incubation with AOPPs enhances TNF-α production by inducing a polarity shift toward inflammatory M1-type macrophages.

Secondly, we investigated whether AOPPs induce adipose inflammation via macrophages. Here, AOPPs were added to RAW264.7 cells, and 12 h later, the conditioned medium (CM) was added to differentiated 3T3-L1 cells. After a further 12 h, the expression of inflammatory cytokines (TNF-α, IL-6) in adipocytes at the mRNA level was evaluated (Figure 3G). The results showed that AOPP-treated CM increased TNF-α and IL-6 expression in differentiated 3T3-L1 cells compared to the control CM or HSA-treated CM groups (Figure 3H,I). Furthermore, TNF-α mRNA expression in adipocytes was up-regulated by the presence of TNF-α (Figure 3H). By contrast, no significant increase in IL-6 mRNA expression was observed in the TNF-α-treated group. These results suggested that AOPPs contributed to adipose inflammation via adipose tissue–macrophage interactions.

### 2.4. Effects of AOPP Overload on Mouse Adipose Tissue

**Figure 4:**
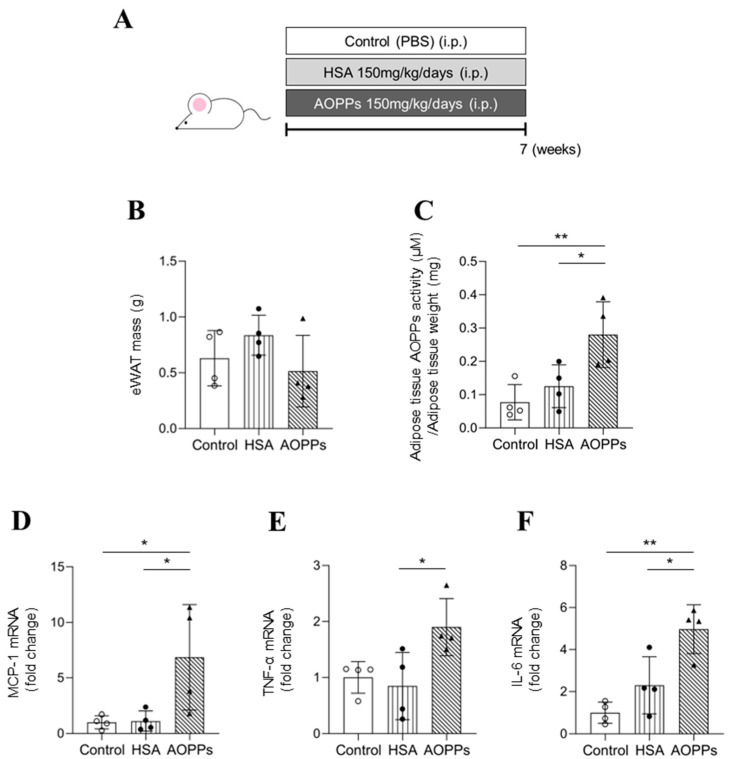
In vivo effect of AOPPs on adipose tissue using AOPP-overloaded mice. (**A**) AOPP-overloaded mice were created by intraperitoneally administering AOPPs at a dosage of 150 mg of protein/kg/day for 7 weeks. In the comparison group, PBS or HSA (150 mg protein/kg/day) was intraperitoneally administered as well. (**B**) The weight of eWAT was examined 7 weeks after administration of AOPPs. (**C**) AOPP levels were measured in the eWAT. (**D**–**F**) After AOPP administration for 7 weeks, mRNA expression of (**D**) MCP-1, (**E**) TNF-α and (**F**) IL-6 in eWAT were measured via real-time qPCR. Data are expressed as the mean ± SEM (n = 4). * *p* < 0.05, ** *p* < 0.01.

To verify the above results in vivo, we evaluated adipose tissue of AOPP-overloaded mice. Here, 4-week-old healthy ICR mice were intraperitoneally injected with AOPPs on a daily basis (150 mg protein/kg/day) for 7 weeks (AOPP-overloaded mice). The comparison group comprised PBS-treated mice (control) or HSA-treated mice with the same protein concentration as that of AOPPs (150 mg protein/kg/day) (Figure 4A). Plasma biochemical parameters for renal function (BUN and SCr) at 7 weeks after AOPP loading were not significantly different from those of the control and HSA-treated groups (Table S3). Although AOPP overload did not alter body weight, epididymal fat mass tended to decrease (Figure 4B). Moreover, AOPP activity in adipose tissue was significantly increased in the AOPP-overloaded group compared to the control or HSA-treated groups (Figure 4C).

The effect of AOPP overload on adipose tissue inflammation was also evaluated. MCP-1 mRNA expression was significantly elevated in the eWAT of AOPP-overloaded mice (Figure 4D). Indeed, mRNA expression levels of TNF-α and IL-6 were also significantly elevated in the eWAT of AOPP-overloaded mice (Figure 4E,F). These results indicate that AOPPs induced adipose tissue inflammation in vivo.

## 3. Discussion

**Figure 5:**
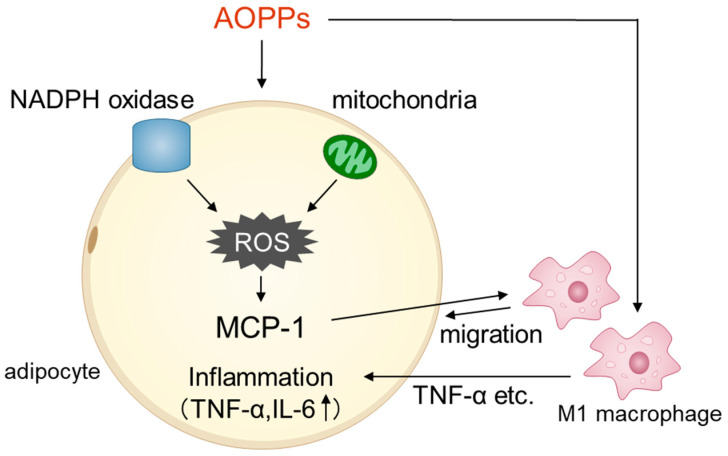
Proposed mechanism for AOPP-induced adipose tissue inflammation. AOPPs enhance MCP-1 secretion via NADPH oxidase- and mitochondria-derived ROS, which induces macrophage migration to adipocytes. AOPPs polarize macrophages into M1-like macrophages and induce TNF-α release. AOPPs then induce macrophage-mediated adipose inflammation.

In this study, we found that AOPPs induced MCP-1 expression in adipocytes through the production of NADPH-oxidase-derived and mitochondria-derived ROS. Moreover, AOPPs were also found to induce the migration of macrophages to adipocytes. AOPPs also up-regulated TNF-α expression by polarizing macrophages to an M1-type polarity, leading to macrophage-mediated adipose inflammation (Figure 5). A previous report demonstrated that uremia resulted in macrophage-mediated adipose inflammation [24]. However, to date, which uremic toxins increase macrophage-mediated adipose inflammation has not been clarified. The results from this study suggest that the uremic toxin AOPPs contribute to macrophage-mediated adipose inflammation.

Based on experiments using co-cultured adipocytes and macrophages, we found that AOPPs up-regulated MCP-1 expression in adipocytes and this was involved in macrophage migration (Figure 2D,E). We also showed that AOPPs induced adipocyte inflammation via macrophages in a series of experiments using a culture medium (CM) of macrophages (Figure 3H,I). Indeed, AOPPs were found to act on macrophages to induce M1-type polarity changes and enhance the release of TNF-α (Figure 3C–F). The addition of TNF-α to adipocytes also increased TNF-α mRNA expression in adipocytes (Figure 3H).

It was previously demonstrated that increased MCP-1 is involved in obesity-related adipose tissue inflammation by acting on macrophage migration and inducing the local proliferation of macrophages [25], which contribute to metabolic abnormalities such as persistent adipose inflammation and insulin resistance [26]. Suganami et al. also reported that TNF-α is a major macrophage-derived paracrine mediator involved in adipose tissue inflammation [27]. During obesity, adipose tissue macrophages polarize to M1-type macrophages, which then release inflammatory cytokines such as TNF-α [28].The infiltrating macrophages interact with adipocytes in a paracrine fashion to further increase the secretion of proinflammatory cytokines [12].This crosstalk between adipocytes and macrophages causes a vicious cycle in obese adipose tissue [29].Taking these previous reports and the present study into consideration, increased AOPPs in CKD could contribute to macrophage migration to adipocytes and its changing polarization then induces adipose tissue inflammation. Recently, Liao et al. reported that AOPPs induced autophagy impairment in macrophages by suppressing the nuclear translocation of transcription factor EB (TFEB) through the activation of the PI3K-AKT-mTOR pathway [30]. Autophagy impairment in macrophages has been shown to induce M1 polarization [31,32]. These findings suggest that the induction of M1-type macrophages by AOPPs may involve autophagy impairment.

Previously, AOPPs were reported to promote ROS production in adipocytes via the activation of NADPH oxidase [33]. Mitochondria as well as NADPH oxidase are known to be intracellular sources of ROS production related to adipose inflammation [34]. However, the effect of AOPPs on mitochondria-derived ROS in adipocytes has not been determined. In this study, we found that in addition to the involvement of NADPH oxidase, mitochondria-derived ROS also contributed to AOPP-induced ROS production (Figure 2A). Mitochondria-derived ROS in adipocytes enhance lipolysis by inducing excessive mitophagy via the NF-κB pathway and increasing inflammatory cytokine expression. Elevated free fatty acids in the blood contribute to hepatic insulin resistance and the progression of type 2 diabetes mellitus [35]. Therefore, AOPPs may also affect systemic metabolic abnormalities during CKD via increased ROS production in adipocytes.

For the cellular uptake of AOPPs, the involvement of CD36 and the receptor for advanced glycation end products (RAGE) have been suggested. Specifically, in renal tubular cells, AOPPs are taken up by CD36, and then mitochondria-derived ROS are released via PKC signaling activation [36]. In chondrocytes, AOPPs induced chondrocyte apoptosis by increasing NADPH-oxidase-derived ROS production after being taken up via RAGE [37]. Adipocytes also express CD36 and RAGE. Kuniyasu et al. reported that oxidized LDL promoted ROS production after being taken up via CD36 in adipocytes, resulting in enhanced PAI-1 expression [38]. Feng et al. reported that RAGE deficiency suppressed MCP-1 expression and macrophage infiltration in adipocytes in a high-fat-diet (HFD)-induced obesity model, indicating that RAGE-mediated signaling might be involved in adipose inflammation and the development of insulin resistance during obesity [39]. Based on these previous findings, it is suggested that CD36 and RAGE could be involved in the uptake of AOPPs into adipocytes. Further investigation is required to verify the involvement of CD36 and RAGE on AOPP-induced adipose inflammation. To this end, experiments using neutralizing antibodies and siRNA will be conducted.

In the present study, AOPPs did not affect the expression of inflammatory cytokines in adipocytes without macrophages (Figure 3A,B). However, Qin Gen Zhou et al. reported that AOPPs induce inflammatory cytokine expression via the NF-κB pathway in adipocytes [36]. These conflicting results may be due to either differences in the albumin (mouse- or human-derived albumin) used in the experiments, or the different oxidants (hypochlorous acid or chloramine-T) employed in AOPP preparation. As a consequence, the properties of the resulting AOPPs may differ between the two studies.

## 4. Conclusions

Here, we show that AOPPs induced oxidative stress and inflammation in adipose tissue via interaction with adipocytes and macrophages. As such, AOPPs represent a promising new therapeutic target for fat atrophy associated with CKD.

## 5. Materials and Methods

### 5.1. Chemicals and Materials

Human serum albumin (HSA) was purchased from the Japan Blood Products Organization (Tokyo, Japan). Diphenylene iodonium (DPI), MitoTEMPO, insulin, dexamethasone and isobutylmethylxanthine were purchased from Sigma-Aldrich (St Louis, MO, USA). Anti-F4/80 monoclonal antibody was purchased from eBioscience (San Diego, CA, USA). Potassium iodide, chloramine T, acetic acid and N-acetyl-L-cysteine (NAC) were purchased from Nacalai Tesque (Kyoto, Japan). 5-(and 6)-chloromethyl-2′,7′-dicholorodihydrofluorescein diacetate (CM-H2DCFDA) and Dulbecco’s phosphate-buffered saline (D-PBS) were purchased from Invitrogen (Grand Island, NY, USA). Dulbecco’s modified eagle medium (DMEM)-high glucose and DMEM-low glucose were purchased from FUJIFILM Wako Pure Chemical Co. (Osaka, Japan). All methods were carried out in accordance with approved guidelines. All experimental protocols were approved by Kumamoto University.

### 5.2. Cell Cultures

Mouse 3T3-L1 fibroblasts were maintained in DMEM-low glucose containing 10% bovine calf serum (GE Healthcare, UK Ltd., Amersham, UK) and supplemented with 1% penicillin/streptomycin. The differentiation of mouse 3T3-L1 fibroblasts to mature adipocytes was performed by exposing post-confluent cells for 2 days to an induction medium. The induction medium consisted of DMEM-high glucose containing 10% FBS (Capricorn Scientific, Ebsdorfergrund, Germany), 1% penicillin/streptomycin, 10 µg/mL insulin, 2.5 µM dexamethasone and 0.5 mM isobutylmethylxanthine. After 2 days, the medium was changed to a maturation medium. The maturation medium consisted of DMEM-high glucose containing 10% FBS, 1% penicillin/streptomycin and 10 µg/mL insulin. The mature medium was exchanged every other day until day 12. Cells were serum-starved for 12 h prior to starting the experiment. RAW264.7 cells were obtained from the RIKEN BRC Cell Bank (Ibaraki, Japan), and were grown in DMEM-high glucose containing 10% FBS and 1% penicillin/streptomycin.

### 5.3. Assay Procedure for AOPPs

The protocol used to determine the level of AOPPs was described in a previous report [16]. In brief, a 200 μL aliquot of the sample was diluted 10-fold in 67 mM phosphate buffer (pH 7.4) and added to a 96-well plate. To each well was added 25 μL of 20% acetic acid and 10 μL of 1.16 M potassium iodide. A standard curve was prepared using chloramine T solution. Absorbance readings at 340 nm were measured immediately after the solution addition using a microplate reader.

### 5.4. Preparation of AOPPs

AOPPs were prepared as described in a previous study [40]. HSA was defatted via treatment with activated carbon. Defatted HSA (300 μM) was incubated with 100 mM chloramine T in 67 mM phosphate buffer (pH 8.0) for 1 h at 37 °C. The oxidation reaction was stopped by dialysis with phosphate-buffered saline (PBS). After dialysis, samples were freeze-dried to prepare AOPPs (194.4 μmol/g protein).

### 5.5. Animal Experiments

All animals were purchased from Japan SLC (Shizuoka, Japan). Animals were housed in a temperature controlled room (21–23 °C) with a 12 h light/dark cycle (light 8 am to 8 pm) and given ad libitum access to food and water. All animal experiments were conducted using procedures approved by the experimental animal ethics committee at Kumamoto University (approval number A2021-021). Adenine-induced renal failure mice (CKD mice) were established using a protocol described in a previous report [41]. C57BL/6NCrSlc mice (male, 6 weeks) were fed CE-2 (normal diet) for 1 week for pre-rearing, and then switched to an adenine powder mixed diet (normal diet supplemented with 0.2% adenine) for 4 weeks. The mice were then switched to CE-2 to rule out any direct adipose tissue effects of adenine, and evaluated after 4 weeks of feeding.

For AOPP-overloaded mice, AOPPs were administered intraperitoneally on a daily basis to 4-week-old male ICR mice for 7 weeks. As a control, PBS or defatted HSA (150 mg protein/kg/day: the same amount of protein as AOPPs) was also administered to male ICR mice (4-week-old) for 7 weeks.

### 5.6. ROS Measurements

3T3-L1 cells were seeded on 96-well plates at 1.0 × 10^4^ cells per well and differentiated into mature adipocytes. After cell differentiation, the cells were washed with PBS and starved with serum-free medium for 12 h before being treated with CM-H2DCFDA for 30 min in D-PBS. After removing the supernatant, cells were incubated with AOPPs, HSA or D-PBS (control) for 1 h. Fluorescence intensity was then measured using a fluorescence plate reader (Synergy H1, Agilent BioTek, Santa Clara, CA, USA) with an excitation and emission of 485 nm and 535 nm, respectively. In the study using inhibitors, cells were incubated with CM-H2DCFDA for 30 min, and then the supernatant was replaced with D-PBS. After the addition of the various inhibitors and incubation for 30 min, AOPPs, HSA or D-PBS was added in the presence of each inhibitor. Fluorescence intensity was then measured as described earlier.

### 5.7. Quantitative RT-PCR

Total RNA was isolated from differentiated 3T3-L1 cells, RAW264.7 cells or adipose tissue using RNAiso Plus (Takara, Tokyo, Japan). The concentration and purity of the extracted RNA was determined from absorbance readings at 260 and 280 nm. A master mix was used to prepare the cDNA from the extracted RNA. Quantitative RT-PCR measurements were then performed. Sequences of the primers used for mRNA detection are given in the supporting information (Table S1). The threshold cycle (Ct) values for each gene amplification were normalized by subtracting the Ct value calculated for GAPDH.

### 5.8. Histological Analysis

Adipose tissues were harvested from the mice and fixed for 48 h with 10% neutral buffered formalin solution at 4 °C. Each tissue sample was processed via paraffin infiltration using a fully automated sealed tissue processor (ASP300S, Leica) and then embedded with paraffin. Sections were cut at a thickness of 5-μm and mounted on glass slides. For the measurement of adipocyte size, sections were stained with hematoxylin and eosin. Quantification was performed from 5 fields of image per sample. Adipocyte size was calculated from the long diameter obtained via microscopy (BZ-X710 microscope; Keyence, Osaka, Japan). For F4/80 staining, the deparaffinized sections were antigen-activated with Histo VT One (Nacalai Tesque) and incubated with anti-F4/80 antibody (1:50) overnight at 4 °C. The sections were then reacted with peroxidase-conjugated anti-rat IgG antibody (Histofine Simple Stain MAX-PO; Nichirei Biosciences, Tokyo, Japan) at room temperature for 30 min, followed by reaction with DAB solution at room temperature for 1.5 min. After counterstaining with hematoxylin, the sections were dehydrated and permanently mounted. Crown-like structures (CLS) were counted with a microscope at a ×20 magnification from 6 images per sample, as described previously [42]. All images were randomly acquired using a BZ-X710 microscope.

### 5.9. Transwell^®^ Assays

Transwell assays were used to detect macrophage migration. 3T3-L1 cells were seeded on 12-well Transwell^®^ plates at 2.0 × 10^5^ cells per well and differentiated into mature adipocytes. After cell differentiation, AOPPs (100 μM) or HSA (protein concentration equivalent to 100 μM of AOPPs) were added to differentiated 3T3-L1 cells, and 48 h later, Transwell^®^ inserts (3-μm pore size) seeded with RAW264.7 (1.0 × 10^4^ cells per well) were inserted and co-cultured for 12 h. Macrophages migrating to the lower layer of the semipermeable membrane of the Transwell^®^ inserts were evaluated via fluorescent immunostaining with anti-F4/80 antibody. The semipermeable membrane was washed once with PBS and cells present in the upper layer of the semipermeable membrane were removed. After cutting the semipermeable membrane along the edges, the cells were fixed by incubating them in 4% paraformaldehyde for 20 min at room temperature. Cells were washed once with PBS, blocked for 1 h at room temperature, and then incubated with rat anti-F4/80 antibody (1:50) overnight at 4 °C. After washing three times with PBS, a secondary antibody reaction was performed with Alexa Fluor647 anti-rat IgG antibody at room temperature for 1 h. The cells were washed three times with PBS and treated with vectashield antifade mounting medium prior to observation under a fluorescence microscope (BZX-710). F4/80 positive areas were quantitated from 5 fields of view randomly selected from each slide. Analysis was performed using a BZ-X Analyzer.

### 5.10. Preparation of RAW264.7-Conditioned Medium (CM)

RAW264.7 cells were cultured in DMEM-high glucose containing 10% FBS and 1% penicillin/streptomycin. The cells were treated with AOPPs or HSA for 12 h and then the medium was collected and centrifuged at 1500 rpm for 5 min. The supernatant was sterilized by filtration through a 0.22 μm filter unit and used as RAW264.7-CM.

### 5.11. Cytokine Production Assay Using ELISA

The culture medium was collected and clarified via centrifugation (1500 rpm, 5 min) prior to assaying. Assay samples were stored at −80 °C. MCP-1 and TNF-α were quantified using ELISA kits (BioLegend, San Diego, CA, USA). Assays were performed according to the manufacturer’s protocol.

### 5.12. Statistical Analyses

The means for two group datasets were compared using the unpaired t test. The means for more than two groups were compared via one-way ANOVA followed by Tukey’s multiple comparison. Probability values of *p* < 0.05 or *p* < 0.01 were considered to be significant.

## Data Availability

Data are contained within the article or Supplementary Material. The data presented in this study are available in [doi of this article].

## Supplementary Materials

The following are available online at https://www.mdpi.com/article/10.3390/toxins15030179/s1, Table S1: Sequence of primers used for mRNA detection. Table S2: Renal function and body weight profile for control or adenine-induced CKD mice., Table S3: Renal function and body weight for control, HSA-overloaded or AOPP-overloaded mice.
