# Supplementary material for: Advanced Oxidation Protein Products Contribute to Chronic-Kidney-Disease-Induced Adipose Inflammation through Macrophage Activation
Source: Toxins (Basel). 2023 Feb 26;15(3):179. doi: 10.3390/toxins15030179 (PMC10059001; doi:10.3390/toxins15030179)
Supplement: Supplementary file 1 [file toxins-15-00179-s001.zip › toxins-2233647-supplementary.pdf]

Supplementary Materials: Advanced oxidation protein products contribute to chronic kidney disease-induced adipose inflammation through macro-phage activation

Nanaka Arimura, Hiroshi Watanabe, Hiromasa Kato, Tadashi Imafuku, Takehiro Nakano, Miyu Sueyoshi, Mayuko Chikamatsu, Kai Tokumaru, Taisei Nagasaki, Hitoshi Maeda, Motoko Tanaka, Kazutaka Matsushita and Toru Maruyama

Table S1. Sequence of primers used for mRNA detection.

Primer Name	Forward Primer Sequence (5′-3′)	Reverse Primer Sequence (5′-3′)
Mouse MCP-1	GATGCAGTTAACGCCCCACT	GAGCTTGGTGACAAAAACTACAG
Mouse TNF-	TAGCCCACGTCGTAGCAAAC	GCAGCCTTGTCCCTTGAAGA
Mouse IL-6	ACAAAGCCAGAGTCCTTCAGAG	TGTGACTCCAGCTTATCTCTTGG
Mouse iNOS	AGTCAACTGCAAGAGAACGGA	GAAGAGAAACTTCCAGGGGCA
Mouse CD206	TTCAGCTATTGGACGCGAGG	GAATCTGACACCCAGCGGAA
Mouse GAPDH	AACTTTGGCATTGTGGAAGG	ACACATTGGGGGTAGGAACA

Table S2. Renal function and body weight profile for control or adenine-induced CKD mice.

	Control	CKD
BUN (mg/dL)	43.3 ± 1.6	119.7 ± 2.2 [a]
SCr (mg/dL)	0.40 ± 0.04	0.69 ± 0.02 [a]
Body weight (g)	27.1 ± 0.2	23.1 ± 0.4 [a]

Data are expressed as the mean ± SE ($n = 5$). [a] $P < 0.01$ compared with control. BUN, blood urea nitrogen; SCr, serum creatinine.

Table S3. Renal function and body weight for control, HSA-overloaded or AOPPs-overloaded mice.

	Control	HSA	AOPPs
BUN (mg/dL)	26.4 ± 1.1	29.8 ± 2.1	35.7 ± 2.4 [a]
SCr (mg/dL)	0.21 ± 0.02	0.23 ± 0.01	0.17 ± 0.04
Body weight (g)	42.9 ± 1.7	44.4 ± 0.9	43.3 ± 1.4

Data are expressed as the mean ± SE ($n = 4$). [a] $P < 0.05$ compared with control. BUN, blood urea nitrogen; SCr, serum creatinine.
